# Effects of a combined lifestyle score on 10-year mortality in Korean men and women: a prospective cohort study

**DOI:** 10.1186/1471-2458-12-673

**Published:** 2012-08-20

**Authors:** Ji Eun Yun, Soyoung Won, Heejin Kimm, Sun Ha Jee

**Affiliations:** 1 Department of Rehabilitation Standard & Policy, Korea National Rehabilitation Research Institute, Seoul, Republic of Korea; 2 Institute for Health Promotion, Yonsei University, Seoul, Republic of Korea; 3 Department of Epidemiology and Health Promotion, Graduate School of Public Health, Yonsei University, Seoul, Republic of Korea

**Keywords:** Lifestyle factor, Mortality, Cohort study, Population attributable risk

## Abstract

**Background:**

Most studies that have evaluated the association between combined lifestyle factors and mortality outcomes have been conducted in populations of Caucasian origin. The objective of this study was to examine the association between combined lifestyle scores and the risk of mortality in Korean men and women.

**Methods:**

The study population included 59,941 Koreans, 30–84 years of age, who had visited the Severance Health Promotion Center between 1994 and 2003. Cox regression models were fitted to establish the association between combined lifestyle factors (current smoker, heavy daily alcohol use, overweight or obese weight, physical inactivity, and unhealthy diet) and mortality outcomes.

**Results:**

During 10.3 years of follow-up, there were 2,398 cases of death from any cause. Individual and combined lifestyle factors were found to be associated with the risk of mortality. Compared to those having none or only one risk factor, in men with a combination of four lifestyle factors, the relative risk for cancer mortality was 2.04-fold, for non-cancer mortality 1.92-fold, and for all-cause mortality 2.00-fold. In women, the relative risk was 2.00-fold for cancer mortality, 2.17-fold for non-cancer mortality, and 2.09-fold for all-cause mortality. The population attributable risks for all-cause mortality for the four risk factors combined was 44.5% for men and 26.5% for women.

**Conclusion:**

This study suggests that having a high (unhealthy) lifestyle score, in contrast to a low (healthy) score, can substantially increase the risk of death by any cause, cancer, and non-cancer in Korean men and women.

## Background

There are many studies that indicate that individual healthy lifestyle factors such as non-smoking, normal weight, moderate alcohol intake, regular exercise, and a healthy diet reduce the risk of various chronic diseases including cancer and cardiovascular disease [1-11]. Cigarette smoking has been identified as the second leading risk factor for death from all causes worldwide [12,13] and being overweight or obese (and possibly underweight) are associated with increased all-cause mortality [4,9]. Physical inactivity and heavy alcohol use are related to the risk of mortality, coronary heart disease, and cancer incidence [1,2,6,10]. Moreover, previous studies have shown that lifestyle modification may prevent or slow the incidence of cancer [14-16]. As such, maintaining a healthy lifestyle is becoming a main focus of today’s society.

A number of studies have shown that there is a significant increase in the incidence of cancer and/or mortality when an individual has several bad lifestyle habits measured by scoring individual lifestyle factors. The Nurses’ Health Study of middle-aged women in the United States implicated a combination of lifestyle risk factors in relation to mortality [17]. In the European Prospective Investigation of Cancer (EPIC)-Norfolk study, lifestyle factors were studied in relation to mortality in both men and women between the ages of 45 and 74 at study initiation [18]. In another study of older individuals in the United States, the risk of pancreatic cancer was found to be significantly lower in participants with combined healthy lifestyle scores. A combined lifestyle score was calculated by summing the scores of the lifestyle factors. These studies have been conducted in specific populations of Caucasian origin [17-20], the elderly [19,21] or in women only [8,17,22]. Few studies have examined the relationship between combined lifestyle factors and mortality in East Asian countries [22,23].

The Korean lifestyle differs from that of Western and European societies. For example, despite the extremely high prevalence of smoking, populations in Korea tend to be leaner and have lower levels of serum cholesterol and perhaps less atherosclerosis than their Western counterparts [24]. Thus, research is needed in this area on ethnic minority populations. Therefore, we evaluated the association between combined lifestyle scores and risk of total and cause-specific mortality in the Korean Cancer Prevention Study.

## Methods

### Study population

The cohort consisted of 70,843 Korean men and women who participated in at least one medical evaluation at the Severance Health Promotion Center between 1994 and 2003. Study participants included people who were over the age of 30 (n = 65,520). To avoid confounding the association of lifestyle factors with mortality due to any pre-existing conditions, we excluded 787 participants who reported having cardiovascular disease and any cancer at or prior to their initial visit. In addition, 4,792 subjects with missing information with regards to smoking status and alcohol intake were also excluded. The final study sample consisted of 59,941 subjects. The mean follow-up period was 10.3 years, through December 31, 2009. The Institutional Review Board of Yonsei University College of Medicine approved this study.

### Data collection

Data were collected from participants who underwent medical examinations from 1994 to 2003. A skilled data entry clerk entered results from questionnaire surveys and medical assessments obtained from participants who underwent examinations at the health promotion center. The examinations were performed according to a standard protocol. Each participant was interviewed using a structured questionnaire to collect the lifestyle data. Data from the baseline interview were used to assess the lifestyle factors of interest. Participants were asked to describe their smoking habit (never smoker, ex-smoker, or current smoker), alcohol consumption (non-drinker or drinker of any amount of alcohol), exercise routine (exercise or non-exercise) as well as other demographic characteristics such as age, gender, and past history of diabetes or hypertension. Body weight and height were measured while participants were wearing light clothing and body mass index (BMI) was calculated based on weight and height information.

### Lifestyle factor scoring

We were interested in lifestyle-related factors that are simple to assess and have been well-studied previously in relation to mortality. We used a binary score for each lifestyle factor. Participants received 1 point for each of the following applicable conditions: current smoker (currently smoking or quit smoking < 10 years ago), heavy daily alcohol use (> 2 drinks for men or > 1 drink for women), overweight or obese (BMI > 25), or physically inactive (physical activity never, rarely, or < 3 times per week), otherwise they received 0 points for each factor. We assigned a lifestyle score 1 ranging from 0 (healthiest) to 4 (unhealthy or least healthy) to each participant by summing the binary scores for each of the four lifestyle factors (i.e. smoking, alcohol use, BMI, and physical activity). For the purposes of data analysis, we merged non-lifestyle risk factor group and one lifestyle risk factor group due to a small number of cases of mortality in the first category. In addition, we performed subgroup analysis on subjects who provided information on fruit and vegetable consumption. In the subgroup analysis for lifestyle score 2, we excluded 18,376 participants with missing information on fruit and vegetable consumption. Lifestyle score 2 ranged from 0 (healthiest) to 5 (unhealthy or least healthy) for each participant by summing the binary scores for each of the four lifestyle factors (i.e. smoking, alcohol use, BMI, physical activity, and fruit and vegetable consumption). The frequency of fruit and vegetable intake was divided into quartiles and calculated as the sum of the number of servings per day of fruits and vegetables consumed according to questionnaire answers (Moon et al., 1980). We considered participants in the highest quartile to be in the low risk category (healthy diet) as far as diet was concerned.

### Outcome variables

Participants were followed-up through December 31, 2009. The principal outcome variables were all-cause mortality, cancer mortality, and non-cancer mortality. Outcomes for mortality were confirmed by matching the information to the death records from the National Statistical Office. In Korea, there are certified medical chart recorders who review and abstract the medical chart and assign discharge diagnosis in a standardized form using WHO (World Health Organization) codes. The recorders also complete death certificates based on the information provided by physicians. A computerized search of the death certificate data from the National Statistical Office in Korea was performed using the unique identification number assigned at birth. Mortality from cancer was defined according to the International Classification of Diseases 10th Revision codes (ICD-10 codes, C00 to C99).

### Statistical analysis

Cox proportional hazards models were used to evaluate the independent and combined effects of lifestyle factors on mortality. All analyses were stratified by sex. We computed hazard ratios (HRs) using Cox proportional hazards modeling to adjust for age. We calculated the population attributable risk (PAR) from the lifestyle score on the risk of mortality [25]. Trend analyses were conducted to test the linearity of the association between the numbers of lifestyle scores and mortality risk. All analyses were conducted using SAS, version 9.1 (SAS Institute Inc., Cary, NC, USA). Statistical significance was determined as *p* < 0.05.

## Results

Over 10.3 years of follow-up, there were 2,398 deaths from all causes (1,591 in men, 807 in women), including 1,108 from cancer (799 in men, 309 in women) and 1,290 non-cancer deaths (792 in men, 498 in women). The general characteristics of the 31,850 male and 28,091 female study participants at baseline are shown in Table 1. Men were younger and had higher blood pressure, total cholesterol, and fasting blood glucose levels than women (P < 0.05 for all). Significantly higher rates of smoking, alcohol intake, and regular exercise were also observed in men compared to women. The percentages of subjects with the healthiest score (0 point) were 0.65% and 1.01% for men and women, respectively. Notably, there was a significant gap between men and women having the worst score (4 points) at 15.3% and 1.6%, respectively.

**Table 1:** General characteristics of the study population

	**Men (n = 31,850)**	**Women (n = 28,091)**
	Mean ± SD	Mean ± SD
Age, y	46.7 ± 10.6	47.7 ± 10.6
Body mass index, kg/m^2^	24.2 ± 2.9	23.5 ± 3.2
Systolic blood pressure, mm Hg	124.5 ± 19.3	121.7 ± 20.9
Diastolic blood pressure, mm Hg	73.1 ± 12.1	72.4 ± 11.9
Total cholesterol, mg/dL	197.0 ± 35.1	196.0 ± 37.5
Fasting blood glucose, mg/dL	98.6 ± 26.0	94.0 ± 22.0
	n (%)	n (%)
Smoking status		
Never smoked	9,298 (29.2)	25,940 (92.3)
Ever smoked	22,552 (70.8)	2,151 (7.7)
Adiposity		
Normal weight (18.5 ≤ BMI < 25)	18,726 (58.8)	18,639 (66.4)
Overweight or underweight	13,124 (41.2)	9,452 (33.7)
Physical activity		
≥ 3 times per week	14,634 (46.0)	9,981 (35.5)
< 3 times per week	17,216 (54.0)	18,110 (64.5)
Alcohol intake		
Light to moderate alcohol intake	1,646 (5.2)	873 (3.1)
Heavier drinking or abstaining	30,204 (94.8)	27,218 (96.9)
Fruit and vegetable consumption		
Healthy diet (highest quartile)	6,917 (30.9)	4,238 (22.1)
Unhealthy diet	15,454 (69.1)	14,956 (77.9)
No. of lifestyle score 1		
0-1	3,511 (11.0)	6,768 (24.1)
2	10,099 (31.7)	13,973 (49.7)
3	13,366 (42.0)	6,899 (24.6)
4	4,874 (15.3)	451 (1.6)
No. of lifestyle score 2		
0-1	1,055 (4.7)	1,384 (7.21)
2	4,014 (17.9)	5,791 (30.2)
3	7,714 (34.5)	8,298 (43.2)
4	7,326 (32.8)	3,470 (18.1)
5	2,262 (10.1)	251 (1.3)

Data are expressed as mean ± SD or percent.

*BMI* body mass index.

Table 2 shows the main effect of each factor on the risk of mortality. In all subjects, after adjusting for the other risk factors, smoking and less physical activity were associated with a statistically significant increased risk of all-cause, cancer, and non-cancer mortality. Alcohol use and BMI were not significant factors, but there was a trend implying that severe alcohol consumption and being overweight or obese were associated with an increased risk of mortality. In addition, we did the analyses with underweight and overweight separately. This study has found no correlation between being overweight with mortality. However, underweight was associated with all cause mortality in men and non-cancer mortality in men and women (data not shown). The estimated PAR for smoking was higher than for other lifestyle factors (data not shown).

**Table 2:** Hazard ratios* (95% confidence intervals) of all cause, cancer, and non cancer mortality

	**All-cause deaths**		**Cancer deaths**		**Non-cancer deaths**	
**Lifestyle factors**	**Men**	**Women**	**Men**	**Women**	**Men**	**Women**
Ever vs. never smoked	1.46 (1.30-1.65)	1.72 (1.34-2.22)	1.64 (1.39-1.94)	1.43 (0.95-2.16)	1.29 (1.09-1.54)	1.96 (1.42-2.70)
Overweight or underweight vs. normal weight (18.5 ≤ BMI < 25)	1.03 (0.92-1.14)	0.98 (0.83-1.15)	0.95 (0.82-1.11)	1.04 (0.81-1.32)	1.12 (0.96-1.31)	0.94 (0.75-1.16)
Physical activity <3 times per wk vs. ≥3 times per wk	1.35 (1.20-1.51)	1.45 (0.60-3.51)	1.28 (1.09-1.49)	1.07 (0.82-1.38)	1.44 (1.22-1.70)	1.69 (1.31-2.19)
Heavier drinking or abstaining vs. light to moderate alcohol intake	1.09 (0.78-1.52)	1.36 (1.14-1.64)	1.19 (0.75-1.91)	3.45 (0.48-24.62)	0.98 (0.61-1.57)	0.96 (0.35-2.58)

* Hazard ratios adjusted for age and other risk factors included in the table.

Table 3 shows the effects of combinations of lifestyle factors on the risk of all-cause, cancer, and non-cancer mortality which increased as the number of risk factors increased. In the data analysis, we merged a risk factor group having none of risk factors and another risk factor group having only one lifestyle risk factor due to a small number of cases of mortality in the first category. Compared to none or one risk factor, the relative risk of a combination of four lifestyle factors was 2.04-fold for cancer mortality, 1.92-fold for non-cancer mortality and 2.00-fold for all-cause mortality in men, and 2.00-fold for cancer mortality, 2.17-fold for non-cancer mortality and 2.09-fold for all-cause mortality in women. This association remained after further adjustment for potential confounder such as education and income (data not shown). The PAR for all-cause mortality for the four risk factors combined was 44.5% for men and 26.5% for women.

**Table 3:** Hazard ratio of incidence and mortality according to combinations of lifestyle risk factors

	**Lifestyle risk factors**				**PAR(%) for having any of the four risk factors**
	**≤1 risk factor**	**2 risk factors**	**3 risk factors**	**4 risk factors**	
***Men***					
**Cancer deaths**					
Cases	66	242	358	108	48.7
HR* (95% CI)	1.00	1.65 (1.26-2.17)	2.29 (1.76-2.99)	2.36 (1.73-3.22)	
HR† (95% CI)	1.00	1.65 (1.23-2.22)	2.13 (1.59-2.85)	2.04 (1.45-2.87)	
**Non-cancer deaths**					
Cases	75	220	336	134	40.4
HR (95% CI)	1.00	1.26 (0.95-1.66)	1.79 (1.38-2.34)	2.56 (1.90-3.45)	
HR (95% CI)	1.00	1.04 (0.77-1.39)	1.34 (1.00-1.78)	1.92 (1.39-2.64)	
**All-cause deaths**					
Cases	141	462	694	242	44.5
HR (95% CI)	1.00	1.45 (1.19-1.76)	2.04 (1.69-2.46)	2.47 (1.99-3.06)	
HR (95% CI)	1.00	1.32 (1.07-1.63)	1.71 (1.39-2.09)	2.00 (1.58-2.52)	
***Women***					
**Cancer deaths**					
Cases	53	133	106	11	17.3
HR (95% CI)	1.00	1.06 (0.77-1.46)	1.37 (0.98-1.91)	2.15 (1.12-4.13)	
HR (95% CI)	1.00	1.03 (0.72-1.48)	1.04 (0.70-1.54)	2.00 (0.93-4.29)	
**Non-cancer deaths**					
Cases	76	215	170	17	33.8
HR (95% CI)	1.00	1.30 (0.96-1.77)	1.80 (1.31-2.46)	2.69 (1.52-4.78)	
HR (95% CI)	1.00	1.27 (0.89-1.81)	1.68 (1.16-2.44)	2.17 (1.01-4.67)	
**All-cause deaths**					
Cases	129	348	276	28	26.5
HR (95% CI)	1.00	1.18 (0.95-1.47)	1.59 (1.26-1.99)	2.43 (1.58-3.73)	
HR (95% CI)	1.00	1.15 (0.89-1.48)	1.35 (1.03-1.77)	2.09 (1.22-3.59)	

* Hazard ratios and population attributable risks adjusted for age.

† Hazard ratios adjusted for age, income, and education.

In addition, we performed subgroup analysis on subjects who provided information on fruit and vegetable consumption. HRs were higher for non-cancer mortality than for cancer mortality in these men and women. HRs for mortality was increasing with the number of lifestyle risk factors in both men and women (p for trend <0.05). Results for participants who provided information on fruit and vegetable consumption were consistent with results of the entire study population (Table 4).

**Table 4:** Mortality hazard ratios according to combinations of lifestyle risk factors among subjects who provided information on fruit and vegetable consumption

	**Lifestyle risk factors**					**P for trend**
	**≤1 risk factor**	**2 risk factors**	**3 risk factors**	**4 risk factors**	**5 risk factors**	
***Men***						
**Cancer deaths**						
Cases	17	66	172	151	40	
HR* (95% CI)	1.00	1.15 (0.67-1.95)	1.99 (1.21-3.28)	2.23 (1.35-3.69)	2.40 (1.36-4.25)	0.0095
HR† (95% CI)	1.00	1.12 (0.62-2.02)	1.91 (1.10-3.32)	1.94 (1.11-3.39)	1.74 (0.91-3.31)	0.0960
**Non-cancer deaths**						
Cases	15	62	138	161	47	
HR (95% CI)	1.00	1.33 (0.71-2.48)	1.83 (1.01-3.32)	2.74 (1.52-4.94)	3.25 (1.71-6.18)	0.0017
HR (95% CI)	1.00	1.04 (0.55-1.97)	1.25 (0.68-2.28)	1.82 (1.00-3.33)	2.10 (1.08-4.06)	0.0106
**All-cause deaths**						
Cases	32	128	310	312	87	
HR (95% CI)	1.00	1.22 (0.82-1.83)	1.92 (1.31-2.81)	2.44 (1.67-3.58)	2.77 (1.81-4.23)	0.0014
HR (95% CI)	1.00	1.08 (0.70-2.39)	1.59 (1.06-2.39)	1.89 (1.25-2.85)	1.93 (1.22-3.06)	0.0091
***Women***						
**Cancer deaths**						
Cases	5	47	65	52	5	
HR (95% CI)	1.00	1.88 (0.75-4.72)	1.69 (0.68-4.20)	2.71 (1.08-6.80)	3.68 (1.06-12.75)	0.0136
HR (95% CI)	1.00	1.86 (0.66-5.23)	1.60 (0.57-4.47)	2.07 (0.72-5.94)	2.96 (0.66-13.35)	0.0313
**Non-cancer deaths**						
Cases	10	49	119	72	8	
HR (95% CI)	1.00	2.15 (0.66-7.03)	3.72 (1.18-11.78)	5.12 (1.60-16.36)	6.93 (1.73-27.84)	0.0002
HR (95% CI)	1.00	2.66 (0.63-11.24)	4.10 (1.00-16.88)	5.85 (1.40-24.43)	8.74 (1.59-48.14)	0.0013
**All-cause deaths**						
Cases	15	96	184	124	13	
HR (95% CI)	1.00	1.98 (0.96-4.09)	2.45 (1.20-5.00)	3.61 (1.76-7.42)	4.90 (1.97-12.23)	0.0015
HR (95% CI)	1.00	2.13 (0.92-4.93)	2.43 (1.06-5.55)	3.32 (1.43-7.69)	4.85 (1.62-14.52)	0.0042

* Hazard ratios adjusted for age.

† Hazard ratios adjusted for age, income, and education.

## Discussion

In this cohort study, over a period of 10 years, we found multiple lifestyle factors to be associated with mortality from cancer, non-cancer, and all causes. The unhealthy lifestyle factors evaluated were each associated with noticeably greater mortality. Also, we found the risk of all-cause, cancer, and non-cancer mortality to be significantly higher in participants who had the highest combined unhealthy lifestyle scores (currently smoking, no physical activity, overweight, and heavier alcohol consumption) compared to participants with the lowest score. By adherence to these four healthy lifestyle guidelines, we estimated a decrease in mortality of approximately 44.5% for men and 26.5% for women due to all causes, 48.7% for men and 17.3% for women from cancer, and 40.4% for men and 33.8% for women for non-cancer mortality. These trends are similar to the results of subgroup analysis on subjects who provided information on fruit and vegetable consumption.

Previous studies have demonstrated individual risks or protective factors which could be used to predict mortality. Each lifestyle factor, such as smoking, alcohol use, diet, BMI, and physical activity has been studied independently in relation to mortality or cancer incidence [17,21]. In this study, the association between alcohol use, BMI and mortality were not significant, but cigarette smoking and physical inactivity were independently associated with an increased risk of mortality. In the Nurses’ Health Study, each lifestyle factor, including heavier drinking, and being overweight, independently and significantly predicted mortality [17]. Long-term regular exercise is known to induce biochemical changes to the blood and heart which increase energy productivity and physical activity as well as prevent coronary heart disease. Moreover, people with high physical activity levels such as sports players have better lipid attributes which implies that they have lower low-density lipoprotein cholesterol and triglyceride levels, and higher high-density lipoprotein cholesterol levels than people who are more sedentary [26,27].

Few studies have evaluated the effects of lifestyle factor combinations on chronic disease [17-21,28]. The findings of the present study are in concordance with direction and magnitude of association of several previous studies. The EPIC-Norfolk Prospective Population Study reported that the combination of four health behaviors predicted a 4-fold difference in total mortality [18]. The Nurses’ Health Study also pointed out that combinations of lifestyle factors are related to mortality; however, only Caucasian women were evaluated [17]. A large cohort study including 450,416 participants found an association between combined healthy lifestyle scores and the risk of pancreatic cancer [21]. Only two Asian studies including a Japanese study and a study of Chinese women showed a significant association between lifestyle-related factors and mortality [22,23]. Most previous studies involving lifestyle factors and chronic diseases were conducted in Caucasians, or the elderly, or in women only; therefore, the Korean Cancer Prevention Study II is of great value due to the fact that an association between lifestyle factors and mortality was confirmed in Korean men and women.

Lifestyle factors, besides having a direct effect on mortality, may also act indirectly. On average, those with a particular negative habit as a lifestyle factor are more likely to have negative habits related to other lifestyle factors and a bad lifestyle pattern in general [29]. For example, physical inactivity may act via other behavioral factors, such as obesity, to affect mortality; as such, one lifestyle factor (e.g., physical inactivity), may be a surrogate for another (e.g., obesity) [30]. Health behaviors are complex and consist of multiple dimensions; thus, using a lifestyle pattern analysis may capture the influence of multiple health behaviors better than analysis based on individual health behaviors [21]. Consequently, multiple lifestyle behavior adjustments such as quitting smoking, increasing physical activity, controlling weight, reducing alcohol consumption, and paying more attention to diet, may lead to decreased mortality. These potential relationships between lifestyle factors and mortality warrant further study.

Our study has several strengths. The present study used a population-based prospective cohort study design and a relatively large sample size. The prospective design minimizes differential misclassification of exposure status. Few investigations have shown the beneficial effects of a healthy lifestyle scores on any-cause, cancer, or cardiovascular mortality in the Asian population. Our research demonstrated the relationship between combined lifestyle factors and total or type-specific mortality. We can see the mortality from all causes, any cancer, and non cancer, simultaneously. Further large prospective studies are required to evaluate the link between lifestyle factors and site-specific cancer incidence, as well as to determine the mechanisms involved. In addition, this study population has their unique characteristics compared to those in previous investigations. Koreans represent one of the world’s most ethnically and genetically homogeneous populations and ideal for association studies. As such, all subjects in this study were of the same ethnicity, Korean [31].

Several potential limitations of this study must also be considered. Participants of this study were not randomly selected. Also, the representativeness of the background population is limited because study subjects were recruited individually as they went to the health promotion center to check their health status. Although the study subjects were a general population, they might have better health-related behavior or higher socioeconomic status than others because they chose to take a routine health examination. Participants may have underreported their smoking status and alcohol consumption in the self-report questionnaires. In addition, lifestyle changes before and after baseline assessments were not taken into account. Also, we did not sufficiently consider diet in scoring lifestyle factors even though diet is a vital factor in determining links between death and disease due to the insufficient data on nutrition survey (such as food frequency questionnaire) related to participant nutrition. Rather than considering the entire dietary quality, we performed a subgroup analysis using data from those who provided information on fruit and vegetable consumption. Finally, when considering the association between lifestyle score and mortality, we were unable to use cause-specific mortality and only considered cancer and non-cancer deaths.

## Conclusions

In conclusion, based upon our findings, combined non-healthy lifestyle factors including smoking, heavier alcohol consumption, obesity, and lack of physical activity may be directly implicated in the increased risk of all-cause, cancer, and non-cancer mortality in Korean men and women. The findings highlight the importance of overall lifestyle modification in disease prevention, and this information should provide incentive to Asian populations to consider healthy lifestyle choices.

## Competing interests

The authors declare that they have no competing interests.

## **Authors’ contributions**

JEY analyzed the data, interpreted the results and drafted the manuscript. All authors contributed to interpretation of results, revised and commented on the manuscript for important intellectual content, and approved the final version. All authors read and approved the final manuscript.

## Pre-publication history

The pre-publication history for this paper can be accessed here:

http://www.biomedcentral.com/1471-2458/12/673/prepub
